# Impact of the Council of State Neurosurgical Societies (CSNS) Fellowship on the Development of Neurosurgical Careers: A Retrospective Study

**DOI:** 10.7759/cureus.103113

**Published:** 2026-02-06

**Authors:** Clayton Rawson, Sam Tenhoeve, Omowumi Oladipo, Ashley Carter, Sawyer Bauer, Brandon Lucke-Wold, Deborah Benzil, Owoicho Adogwa, Akshay Sharma, Michael Karsy

**Affiliations:** 1 Neurological Surgery, Noorda College of Osteopathic Medicine, Provo, USA; 2 Neurological Surgery, University of Utah, Salt Lake City, USA; 3 General Surgery, University of Arizona College of Medicine - Phoenix, Phoenix, USA; 4 Neurosurgery, University of Florida, Gainesville, USA; 5 Neurological Surgery, Cleveland Clinic, Cleveland, USA; 6 Neurological Surgery, University of Texas Southwestern Medical Center, Dallas, USA; 7 Neurological Surgery, University of Michigan, Ann Arbor, USA

**Keywords:** council of state neurosurgical societies, csns, h index, leadership, neurosurgery, organized neurosurgery, socioeconomic

## Abstract

The Council of State Neurosurgical Societies (CSNS) has sponsored annual residents who participate nationally in a socioeconomic fellowship that provides full voting privileges within the CSNS, offers early exposure to organized neurosurgery, and can lead to larger leadership roles. We sought to examine the impact of the CSNS fellowship on the development of neurosurgical careers. All prior CSNS fellows were evaluated based on the location of residency and medical school, formal mentorship, number of publications, H-index, academic versus private practice, subspecialty, gender, and leadership roles in neurosurgery. A retrospective analysis of CSNS fellows was performed using publicly available data.

Among 307 fellows, 108 (35.1%) and 158 (51.5%) entered academic and private practice, respectively. Regarding educational background, 300 (97.7%) held a Doctor of Medicine (MD) degree, 22 (7.2%) held an MD/Doctor of Philosophy (PhD), and 46 (15%) held an MD with an additional degree, including a Master of Business Administration (MBA), Master of Public Health (MPH), Juris Doctor (JD), or Master of Science (MS). In comparison, 7 (2.3%) held a Doctor of Osteopathic Medicine (DO) degree.

With respect to geographic distribution, 184 (60%) of fellows completed residency training on the East or West Coasts of the United States. In terms of scholarly productivity, 214 (70%) had an average H-index of 17.6, and the average number of publications was approximately 77. Subspecialty training was common, with 242 (78.7%) identified as subspecialists. Spine was the most common subspecialty (90, or 29.3%), followed by cerebrovascular (37, or 12%), pediatric (29, or 9.4%), skull base (25, or 8.1%), and neuro-oncology (24, or 7.8%). Peripheral nerve surgery was the least common subspecialty (4, or 1.3%).

CSNS fellows represent a diverse subsection of neurosurgery, with a strong propensity toward academic, leadership-oriented practices. This subset of neurosurgeons demonstrated substantial publication output and scholarly impact. Overall, to our knowledge, this is the first study to demonstrate the potential impact of formalized exposure to organized neurosurgery in fostering future neurosurgical leaders.

## Introduction

The Council of State Neurosurgical Societies (CSNS) represents a compilation of individual state neurosurgical societies, meeting biannually to address a wide array of neurosurgical issues, including socioeconomics, workforce development, billing/coding, and policy-making [1]. Established to serve as a forum for neurosurgeons to influence healthcare policy and socioeconomic issues affecting the field, the CSNS plays a pivotal role in shaping the practice landscape for neurosurgeons across the United States.

In 1999, the CSNS launched a unique fellowship opportunity inviting neurosurgical residents to participate, marking the first structured effort to engage residents in leadership in the socioeconomic aspects of neurosurgery [1,2]. Unlike many fellowships that primarily emphasize clinical mastery, the CSNS fellowship was specifically developed to cultivate excellence in leadership, mentorship, and innovation, with a particular focus on socioeconomics and health policy. The fellowship aims to enhance residents' understanding of the socioeconomic determinants of neurosurgical patient care while also advancing their competencies in medical knowledge, practice-based learning and improvement, interpersonal and communication skills, professionalism, and systems-based practice [1,2].

While the CSNS fellowship provides a valuable opportunity for residents to engage with the socioeconomic aspects of neurosurgery, it is crucial to assess the long-term efficacy of dedicating a year to such specialized education. Therefore, this study aims to evaluate the career paths of previous CSNS fellows, specifically examining their pathways to the fellowship, academic productivity following the fellowship, and leadership positions held throughout their careers. By analyzing these factors, we hope to gain a clearer understanding of how participation in the CSNS fellowship influences professional development and contributes to the broader field of neurosurgery. As subspecialty training and fellowship pathways play an increasingly central role in shaping neurosurgical practice and leadership, evaluating structured fellowship experiences, such as the CSNS program, is essential to understanding their long-term impact on career development and workforce composition.

## Materials and methods

Study group

A retrospective analysis of CSNS fellows was performed using publicly available data from the CSNS website (https://csnsonline.org). Eligibility for the CSNS Socioeconomic Fellowship requires that applicants be members of either the American Association of Neurological Surgeons (AANS) or the Congress of Neurological Surgeons (CNS), with fellows selected annually during the spring meeting of the CSNS. Applicants are typically neurosurgical residents interested in the socioeconomic aspects of practice and in participating in CSNS activities [1]. The CSNS website contains lists of fellows, including year of involvement, the U.S. quadrant of participation, and associated mentors. Data from the inception of the CSNS fellowship program, spanning the years 1999 to 2024, were obtained. Data collected on fellows included region, medical school, residency, degrees, publications, H-index, nature of workplace (academic or private), subspecialty, gender, and fellow year. The region included four quadrants of the U.S.: northeast, northwest, southeast, southwest, and military. Graduate degrees were noted, including the distinction between Doctor of Medicine (MD) and Doctor of Osteopathic Medicine (DO) residents. Academic practice was determined by whether a surgeon worked in a defined residency training program or academic research environment, and by whether an academic title was held. Subspecialties were defined as the fellow’s primary area of practice, as listed on their faculty biography website.

Data analysis

The distribution of residency programs enrolling a CSNS fellow was analyzed. H-index and number of publications were taken from Google Scholar, Scopus, or personal websites. Publication metrics for fellows included the total number of publications on PubMed, but did not include publications from non-PubMed websites. For non-graduated fellows/residents, career subspecialization and time of practice were tabulated when possible, based on individual residency and biography webpages. Missing data among participants were not entered. Data and statistical analysis were carried out using Excel (Microsoft® Corp., Redmond, WA, USA) and IBM SPSS Statistics for Windows, Version 27 (Released 2019; IBM Corp., Armonk, NY, USA).

## Results

Study group demographics

Over a 25-year period, a total of 307 fellows participated in the CSNS fellowship program (Table 1). The cohort was predominantly male, 272 (88.6%). The program maintained a relatively consistent annual intake of fellows, with most years featuring 13 fellows distributed across four geographic quadrants (Northeast, Northwest, Southwest, and Southeast), including a military category (first included during the 2006-2007 fellowship year). The representation of women remained disproportionately low throughout the study period. Three CSNS fellows passed away, one lost their medical license, and a few fellows repeated the fellowship, with two repeating twice and one repeating three times.

**Table 1:** Demographic data for CSNS fellows (with percent vs total number of fellows) DO: Doctor of Osteopathic Medicine; MBA: Master of Business Administration; PhD: Doctor of Philosophy; MS: Master of Science; MPH: Master of Public Health; CSNS: Council of State Neurosurgical Societies

Variable	Number (%)
Total Numbers of Fellows	307 (100.0%)
Sex	
Male	272 (88.6%)
Female	35 (11.4%)
Quadrants	
Military	17 (5.5%)
Northeast	77 (25.1%)
Northwest	71 (23.1%)
Southeast	66 (21.5%)
Southwest	73 (23.8%)
Academic Degrees	
MD	300 (97.7%)
DO	7 (2.3%)
MBA	23 (7.5%)
PhD	22 (7.2%)
MS	26 (8.5%)
MPH	21 (6.8%)
Clinical Subspecialty	
Cerebrovascular	37 (12%)
Functional	30 (9.7%)
Neuro-oncology	24 (7.8%)
General	71 (23.1%)
Pediatric	29 (9.4%)
Peripheral nerve	4 (1.3%)
Skull base	25 (8.1%)
Spine	90 (29.3%)
Trauma	5 (1.6%)
Type of Practice	
Academic	108 (35.1%)
Private	158 (51.5%)
In residency/fellowship	41 (13.4%)

Medical school and residency distribution

The 307 fellows came from 112 medical schools and 90 different residencies. Most medical schools (80/112, or 80%) had one CSNS fellow. The remaining 11% (10/112) each had six or more fellows, with Vanderbilt being the largest, with 12 fellows (Table 2). Most residencies (82/90, or 92%) had just one CSNS fellow, with just 8% (8/90) sending six or more fellows. Cleveland Clinic sent the most, with 13 (4.2%) (Table 3).

**Table 2:** Medical schools from which the most CSNS fellows graduated CSNS: Council of State Neurosurgical Societies

Medical School	Number (%)
Vanderbilt	12 (3.9%)
Case Western Reserve	10 (3.2%)
University of California, San Diego	10 (3.2%)
Georgetown	9 (2.9%)
Harvard	9 (2.9%)
John Hopkins	8 (2.6%)
Stanford	7 (2.2%)
Columbia	7 (2.2%)
Yale	7 (2.2%)
Louisiana State University	6 (1.9%)

**Table 3:** Residencies with the most CSNS fellows CSNS: Council of State Neurosurgical Societies

Residency	Number (%)
Cleveland Clinic	13 (4.2%)
Barrow	10 (3.2%)
Stanford	10 (3.2%)
University of Florida	10 (3.2%)
Penn State	8 (2.6%)
University of Pittsburgh	6 (1.9%)
Wayne State	6 (1.9%)

Academic output and impact

**Figure 1:**
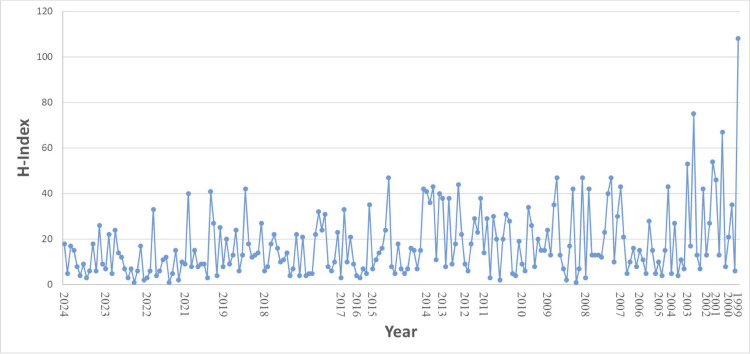
H-index of CSNS fellows by year A graph of CSNS fellows’ current (2024) H-indexes is shown by year of participation in the program. The range of H-index for fellows was between 1 and 109, with a mean of 17.6. CSNS: Council of State Neurosurgical Societies

**Figure 2:**
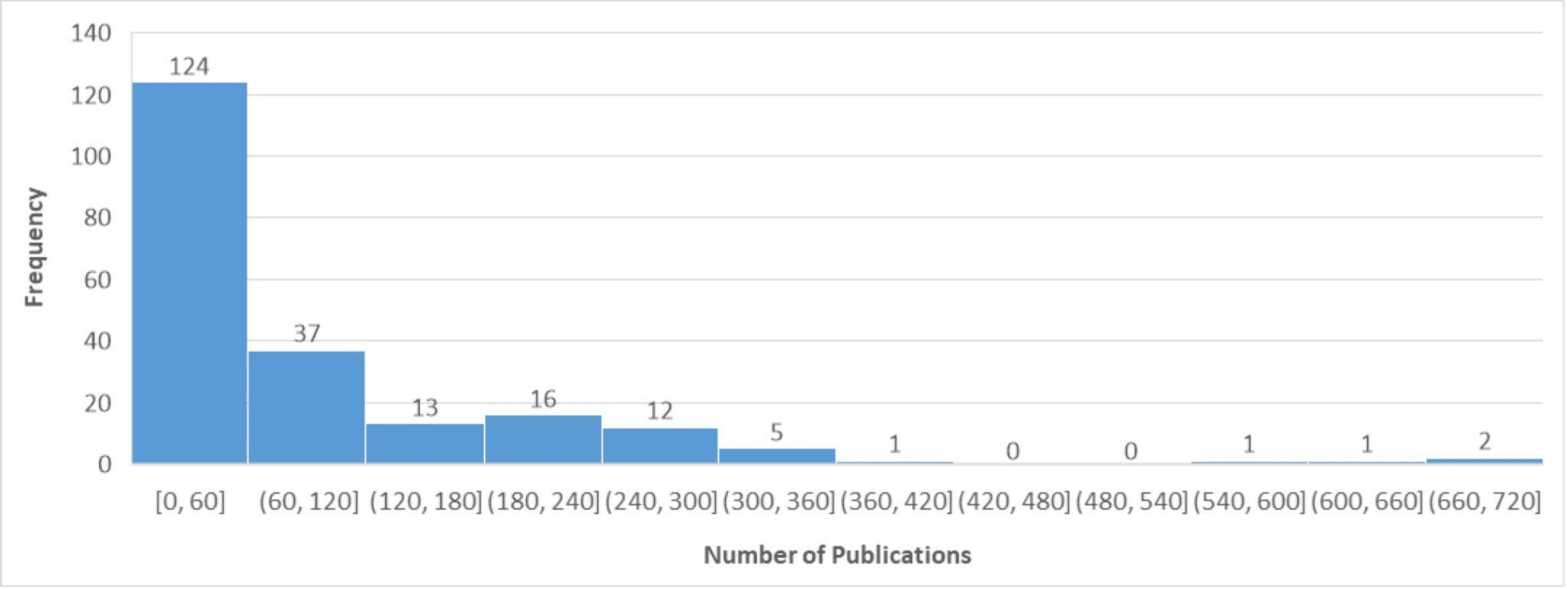
Frequency and number of publications for CSNS fellows A histogram of publication numbers for all CSNS fellows shows that most fellows developed between 0 and 60 career manuscripts, with decreased numbers of individuals having a triple-digit number of published papers. The range of publications for fellows was between 1 and 683, with a mean of 77. CSNS: Council of State Neurosurgical Societies

Publication records for 44 fellows (15%) and H-index for 99 fellows (32%) could not be located. The H-index ranged from 1 to 108, with an average H-index of 17.6 (Figure 1). The average H-index for academic fellows was 19 and ranged from 3 to 108. The average H-index for private fellows was 15 and ranged from 1 to 40. For fellows with available data, the number of publications per fellow ranged from 1 to 683, with an average of approximately 77 publications (Figure 2). Publications and H-index grew significantly over the course of the program, with fellows near the beginning of the program having significantly higher publication numbers and a higher H-index on average than those in the earlier years of the fellowship.

Career paths and additional qualifications

Career choices among the fellows outside of residency and fellowship reveal a preference for private practice over academic positions, with 158 fellows (51.5%) entering private practice and 108 fellows (35.1%) pursuing academic careers. A small portion of the cohort, 41 (13.4%), could not be classified due to still being in residency or fellowship. Regarding additional subspecialization, 70 fellows (22%) did not pursue any further training, while 35 fellows (12%) completed two or more clinical subspecialty fellowships. Spine was the most common clinical fellowship completed, 90 (29.3%), followed by pediatric, 29 (9.4%), cerebrovascular, 37 (12%), neuro-oncology, 24 (7.8%), and skull-base, 25 (8.1%). Peripheral nerve surgery was the least common fellowship, 4 (1.3%) (Table 1).

The vast majority of fellows, 300 (97.7%), held MD degrees, while a smaller percentage, 7 (2.3%), held DO degrees. Additionally, a significant number of fellows pursued advanced degrees beyond their medical training, including 22 (7.2%) Doctor of Philosophy (PhD), 23 (7.5%) Master of Business Administration (MBA), 21 (6.8%) Master of Public Health (MPH), and 1 Juris Doctor (JD).

## Discussion

Study findings

The CSNS resident fellowship offers unique exposure to the socioeconomic aspects of neurosurgical care, along with early, formalized exposure to organized neurosurgery in residency. The outcomes of the fellowship have not yet been evaluated. This study provides data on the career trajectories and academic accomplishments of 307 fellows over 25 years, discussing critical outcomes such as gender representation, publication records, career choices, and additional qualifications outside of neurosurgery. Overall, significant future career publications, clinical subspecialization, and academic involvement may be seen in participants of the CSNS fellowship.

Gender representation

A total of 272 (88.6%) of fellows were men, similar in distribution to overall U.S. neurosurgery residencies [3-5]. Women are significantly underrepresented in neurosurgery, a field traditionally dominated by men. This study further underscores the gender disparities among CSNS fellows, aligning with broader research findings that highlight the persistent challenges faced by women in neurosurgery. Despite recent efforts to promote gender diversity, the proportion of female neurosurgeons remains low, with women comprising less than a tenth of the field in many regions [4-6]. Barriers such as implicit biases, lack of female role models, and limited mentorship opportunities contribute to these disparities [4-6]. Addressing this ongoing gender disparity is crucial for enriching the field of neurosurgery with diverse perspectives and improving patient care through a broader range of insights and experiences. Medical schools and residency programs can begin to address this through targeted recruitment efforts, structured mentorship programs, and initiatives aimed at reducing gender bias within the specialty [7,8].

Fellow publication variability

The academic contributions made by fellows in this study varied significantly. One fellow published 683 career papers, while another published only one, with the average publication count standing at approximately 76, and an average H-index of 17.6. Interestingly, 44 fellows could not be located in publication databases, suggesting potential gaps in data tracking or career paths that do not prioritize academic publishing, such as private practice or non-clinical careers. Existing literature indicates that publication productivity, as measured by metrics like the H-index, is strongly correlated with academic advancement and leadership positions within neurosurgery [9-11]. Higher publication counts during residency and fellowship have been shown to correlate with pursuing an academic career, as prolific publishing often serves as a proxy for research impact, and is linked to increased opportunities for securing academic appointments, grants, and speaking engagements at conferences [11,12]. However, for some fellows, career paths that emphasize clinical practice over research may not necessarily require prolific publishing, reflecting the diverse career aspirations within the field [12,13]. Many fellows enter private practice rather than pursue academic careers, a choice often driven by considerations such as work-life balance, financial incentives, and personal fulfillment [14-16]. These choices may also indicate a trend toward specialization or a need for additional training to meet specific career goals.

Fellow educational background

The educational backgrounds of the fellows provide further insights into career trajectories, with the vast majority holding MD degrees (300, or 97.7%), and a smaller percentage holding DO degrees (7, or 2.3%). Additionally, a significant number of fellows pursued advanced degrees beyond their medical training, such as MBAs, MPHs, or PhDs, suggesting an appreciation for the value of additional knowledge and skills in advancing neurosurgical careers. Research has shown that neurosurgical fellows with certain advanced degrees, like a Master of Science (MS) or PhD, are more likely to practice within academia compared to those with other master’s-level degrees [17,18]. Studies have demonstrated that additional qualifications can enhance career flexibility and impact in academic and leadership roles, equipping neurosurgeons with a broader skill set that includes healthcare management, research, and education [18,19]. For example, an MBA may help neurosurgeons navigate administrative roles or private practice management, while a PhD can bolster research credentials and contribute to academic prestige [17-19]. The presence of these advanced degrees suggests a trend toward diversification of expertise among neurosurgeons, allowing them to contribute to the field in more versatile and impactful ways and to meet the evolving demands of modern neurosurgical practice.

Importance of fellowship programs

Fellowship programs are a critical component of medical education, especially for surgical subspecialties, as they provide an opportunity for concentrated skill development and advanced training beyond the general residency experience [20,21]. These programs are instrumental in bridging the gap between residency and independent practice, offering fellows the chance to refine their surgical skills, gain specialized knowledge, and develop professional networks [22,23]. Beyond technical training, fellowships also foster the growth of autonomy, leadership, and decision-making abilities, which are essential for successful practice in high-stakes environments such as neurosurgery [23-25]. Furthermore, fellowships often emphasize the importance of education and mentorship, equipping trained physicians to become effective educators to peers, residents, and patients, thereby enhancing the overall educational environment within their institutions [23,25]. This mentorship and exposure to diverse educational approaches can significantly influence the fellows' teaching philosophies and methods, contributing to the broader academic and clinical culture.

Fellowships not only provide specialized training but also allow physicians to shape their own personal practice under the guidance of experienced mentors, allowing them to develop unique areas of expertise and niche practices that align with their professional goals and interests [12,14,24]. This personalized mentorship is a distinguishing feature of fellowship training, promoting the development of tailored clinical and research skills that may not be fully addressed in standard residency programs [1,4,23]. Evaluating the long-term impact of fellowship training, including examining the subsequent careers of graduates, is essential to understanding the multifaceted outcomes of these programs. Such assessments provide valuable insights into how fellowship training influences career trajectories, academic productivity, leadership roles, and contributions to the field of neurosurgery.

Limitations

This study, while providing valuable insights into the career trajectories and academic accomplishments of 307 fellows over 25 years, has several limitations. First, the reliance on publication databases to measure academic output may not fully capture the contributions of all fellows, as evidenced by the inability to locate 44 fellows in these databases. This could be due to name changes, career paths that do not prioritize publishing, or gaps in database coverage. Additionally, the study does not account for the potential influence of institutional support, personal circumstances, or regional differences, which may affect fellows' career choices and academic productivity. The gender disparity observed may also be influenced by factors not explored in this study, such as mentorship opportunities, work-life balance, or systemic biases within the field of neurosurgery. The impact of the CSNS fellowship on further organized neurosurgical leadership opportunities and success remains to be established. Challenges remain in identifying the leadership roles that CSNS fellows serve in their careers and the path from the CSNS experience to other potential leadership positions. Lastly, the difference in the impact of the CSNS fellowship and clinical fellowships on overall career trajectories remains to be better evaluated.

Future work

Future research should aim to address the limitations identified in this study by incorporating more comprehensive tracking methods to capture a fuller spectrum of fellows' career outcomes. Longitudinal studies that follow fellows from their training through various stages of their careers could provide deeper insights into how their choices evolve over time and the factors that influence these decisions. Additionally, future studies should explore the underlying causes of gender disparities in neurosurgery, including the role of mentorship, institutional policies, and bias toward CSNS fellows from osteopathic and foreign medical schools. Expanding the scope to include comparisons with other neurosurgical programs or related medical specialties could also help identify broader trends and inform strategies to promote diversity and inclusion across the field. Beyond North America, significant disparities remain in access to advanced neurosurgical subspecialty training in low- and middle-income countries, where limited fellowship opportunities constrain workforce development and clinical capacity. Structured international fellowship collaborations may represent one avenue to disseminate advanced expertise and promote global neurosurgical equity [26]. Moreover, research into the motivations behind the pursuit of advanced degrees and the impact of interdisciplinary training on career success would provide valuable guidance for educational institutions aiming to prepare future neurosurgeons for a rapidly evolving medical landscape.

## Conclusions

This study highlights the significant accomplishments of CSNS fellows as a potential source of future leaders in neurosurgery. The results support a selected group of individuals already within the field of neurosurgery. Trends in gender, publication numbers, and practice patterns were analyzed. These data can be helpful in improving equity for neurosurgical opportunities for all residents and in supporting career development in formalized ways within neurosurgery. The varied career choices of CSNS fellows, and their continued impact, reflect the diversity of opportunities for residents to generate a community impact, as well as future research that can further elucidate the factors shaping the careers of neurosurgical professionals.
